# 抗人T细胞猪免疫球蛋白治疗重型再生障碍性贫血患者的药物代谢动力学研究

**DOI:** 10.3760/cma.j.issn.0253-2727.2022.04.006

**Published:** 2022-04

**Authors:** 丽萍 井, 莉 张, 康 周, 广新 彭, 洋 李, 慧慧 樊, 蕾 叶, 园 李, 建平 李, 琳 宋, 文睿 杨, 凤奎 张

**Affiliations:** 中国医学科学院血液病医院（中国医学科学院血液学研究所），实验血液学国家重点实验室，国家血液病临床医学研究中心，天津 300020 State Key Laboratory of Experimental Hematology, National Clinical Research Center for Blood Diseases, Institute of Hematology & Blood Diseases Hospital, Chinese Academy of Medical Sciences & Peking union Medical College, Tianjin 300020, China

**Keywords:** 贫血，再生障碍性, 抗人T细胞免疫球蛋白, 药代动力学, Anemia, aplastic, Antithymocyte globulin, Pharmacokinetics

## Abstract

**目的:**

研究抗人T细胞猪免疫球蛋白（p-ATG）在重型再生障碍性贫血（SAA）患者的药物代谢特点。

**方法:**

2017年2月至2017年12月纳入接受p-ATG联合环孢素A（CsA）免疫抑制治疗的SAA患者，p-ATG剂量为20 mg·kg^−1^·d^−1^，持续12 h静脉给药，连续5 d。应用三抗体夹心ELISA方法检测p-ATG血药浓度，药代动力学分析软件拟合，计算相关参数并绘制药物代谢曲线。随访记录不良事件并评估治疗后6个月血液学反应。

**结果:**

入组16例接受p-ATG治疗的SAA患者，女8例，男8例，中位年龄22（12～49）岁，中位体重62.5（37.5～82.0）kg。其中14例可进行p-ATG药代动力学评价。p-ATG在体内分布为二室模型，平均药物浓度峰值时间（T_max_）为（5.786±2.486）d，平均峰浓度（C_max_）为（616±452）mg/L，平均半衰期（T_1/2_）为（10.479±8.242）d。平均药物浓度时间曲线下面积［AUC_（0-t）_］为（5.807±3.236）mg/L·d。14例患者治疗后6个月8例获得血液学反应，有效组与无效组患者AUC_（0-t）_分别为（7.50±3.26）mg/L·d对（4.50±2.18）mg/L·d，C_max_分别为（627±476）mg/L对（584±382）mg/L。

**结论:**

连续5 d输注后p-ATG血药浓度达峰值，后缓慢下降，半衰期10.479 d，用药后60 d体内检测到残存药物浓度。尚不能得出药物代谢与疗效及不良反应的关系。

## Abstract (en)

**Objective:**

To study the metabolic characteristics of anti-human T-cell porcine immunoglobulin（p-ATG）in patients with severe aplastic anemia（SAA）.

**Methods:**

For patients with SAA treated with p-ATG combined cyclosporine A（CsA）immunosuppressants between February 2017 and December 2017, the p-ATG dose was 20 mg·kg^−1^·d^−1^ over 12 h of intravenous administration for 5 consecutive days. The blood concentration of p-ATG was detected by the three-antibody sandwich ELISA method, the pharmacokinetic analysis software was fitted, and the second-chamber model method was used to calculate the pharmacokinetic parameters and plot the pharmacokinetic curve. Adverse events were recorded and the hematologic reactions were determined at 6 months after treatment.

**Results:**

Sixteen patients with SAA treated with p-ATG were enrolled, including 8 females and 8 males, with a median age of 22 years（range, 12 to 49 years）and a median weight of 62.5 kg（range, 37.5 to 82.0 kg）. The pharmacokinetics of p-ATG could be evaluated in 14 cases. p-ATG is distributed in vivo as a two-chamber model, with an average drug concentration peak（T_max_）of（5.786±2.486）days, a peak concentration（C_max_）of（616±452）mg/L, and a half-life of（10.479±8.242）days. The area under the drug time curve（AUC）was（5.807±3.236）mg/L·d. Six months after treatment, 8 of 14 patients received a hematologic response; the AUC（0-t）of the effective group and ineffective groups was（7.50±3.26）mg/L·d *vs*（4.50±2.18）mg/L·d, and the C_max_ was（627±476）mg/L *vs*（584±382）mg/L, respectively.

**Conclusion:**

The plasma concentration of p-ATG reached a peak after 5 days of continuous infusion, and then decreased slowly, with a half-life of 10.479 days, and the residual drug concentration was detected in the body 60 days after administration. A relationship between drug metabolism and efficacy and adverse reactions could not be determined.

抗胸腺/淋巴细胞球蛋白（ATG/ALG）联合环孢素A（CsA）的免疫抑制治疗（IST）是不适合或者无法耐受造血干细胞移植（HSCT）的重型再生障碍性贫血（SAA）患者的首选治疗。抗人T细胞猪免疫球蛋白（p-ATG）是我国目前唯一自主生产的此类产品，临床应用该药物治疗SAA患者获得较好疗效，但目前国内外尚未见有关p-ATG治疗SAA的药代动力学报道。近期我们以接受p-ATG联合CsA治疗的SAA患者为研究对象，监测p-ATG的药物浓度变化，了解其药代动力学特性。

## 病例与方法

1. 方案设计：接受IST的SAA患者为中国医学科学院血液病医院住院患者，药物浓度检测单位为中国医药集团武汉生物制品研究所。依据赫尔辛基宣言原则及GCP规范，研究获得我院伦理委员会批准（批件号：ΠT2014015-EC-2），试验前患者本人或其监护人均签署知情同意书。

2. 受试对象：纳入标准：2017年2月至2017年12月中国医学科学院血液病医院贫血诊疗中心住院，参照诊断标准[1]临床诊断为获得性SAA，按照《再生障碍性贫血诊断与治疗中国专家共识（2017年版）》[2]原则，因年龄或供者受限无法接受HSCT，拟接受IST的患者；ECOG评分≤2分；接受各项生化、心电图、超声、骨髓等诊断及筛查评估；患者本人或监护人签署知情同意书。排除标准：先天造血衰竭；患者合并无法控制的严重感染；合并肝肾心等重要脏器功能损伤；合并恶性肿瘤；妊娠或者哺乳期女性。随访至2018年12月，中位随访17（12～22）个月。

3. p-ATG给药方法与样品采集：p-ATG为中国武汉生物制品研究所产品，规格为每支250 mg。给药方案：20 mg·kg^−1^·d^−1^×5 d，皮试阴性后每天连续匀速静脉泵滴，持续12 h输注完毕。另一静脉通路按照1 mg/kg剂量泼尼松，换算为静脉短效氢化可的松（大约总量1/3），剩余剂量换算为静脉地塞米松输注，以预防即刻输注反应。开始p-ATG输注前采集静脉血为0 h，用药过程中观察记录患者的生命体征及药物的不良反应，如发生严重不良事件，立即暂停p-ATG输注，并采取急救措施。整个用药过程患者应用心电监护，并在医护人员的密切监护下层流病房内进行。

接受p-ATG治疗开始至停药后60 d连续不同时间点于用药过程中及用药后采集血样标本，时间点分别为第1天0、4、8、16 h，第2、3、4、5天（用药期间）静脉p-ATG输注前，第6、7、10、15、20、25、35、45、55、65天（停药后）晨起空腹采集静脉血标本。室温下静置30 min，24 °C离心10 min，分离血清，置−70 °C冰箱冷冻保存待测。

4. 血药浓度检测：采用三抗体夹心酶联免疫吸附（ELISA）方法，参照武汉生物制品研究所p-ATG检测试剂盒说明书进行血药浓度检测。应用兔抗猪IgG（美国Santa Cruz公司）包板，胎牛血清封闭；加入样品或对照100 µl，37 °C温浴后洗板5次；然后加入HRP标记鼠抗猪IgG（美国Santa Cruz公司）100 µl，37 °C温浴后洗板5次；再加入显色液反应15 min后，加入终止液终止反应；读取450 nm处吸光度（*A*）值，选择4参数方程式对阳性对照进行回归，计算获得待测样品浓度。实验重复2次取平均值。

5. 药代动力学模型的建立：采用DAS 3.1.4药代动力学分析软件进行拟合，确定权重和房室模型，计算药物代谢动力学参数，包括半衰期（T_1/2_），血药浓度-时间曲线下面积（AUC）、分布容积（V）和清除率（CL）等。AUC用梯形法计算，峰浓度（C_max_）、药物浓度达峰时间（T_max_）用实测值，并利用相关参数绘制药代曲线。

6. 疗效标准：参照中华医学会血液学分会制定的AA疗效标准[2]，根据外周血常规恢复程度、是否依赖血制品输注等指标，将疗效分为完全缓解（CR）、部分缓解（PR）、未缓解（NR），CR、PR共同计入血液学反应率。

7. 不良反应及各项临床指标观测：所有患者在IST期间均密切观察其临床症状体征，包括输注p-ATG期间的即刻不良事件及输注后3周内的血清病反应事件。并且在治疗期间监测淋巴细胞绝对值，观察其与药物浓度的相关性。治疗后监测血液骨髓相关指标，于治疗后6个月进行疗效评价，观察药代动力学指标与疗效的关系。

## 结果

1. 入组患者一般情况：共纳入16例SAA患者，其中女8例，男8例，中位年龄22（12～49）岁，中位身高为162（130～170）cm，中位体重为62.5（37.5～82.0）kg，中位身体质量指数（BMI）为23.5（18.8～33.5）。其中SAA 13例，极重型再生障碍性贫血（VSAA）3例，中位外周血淋巴细胞绝对计数（ALC）为1.84（0.52～3.07）×10^9^ /L（表1）。其中2例患者因其他原因未能按方案完成p-ATG治疗，延长了用药时间，虽其药物浓度及代谢参数也进行了相应检验和安全性数据收集，在数据统计分析过程中仅保留安全性数据，其药代数据未予采用。

**表1:** 16例入组抗人T细胞猪免疫球蛋白药代动力学检测SAA患者一般情况

例号	性别	年龄（岁）	诊断	体重（kg）	身高（cm）	BMI	治疗前血常规					
							ALC（×10^9^/L）	WBC（×10^9^/L）	HGB（g/L）	ANC（×10^9^/L）	PLT（×10^9^/L）	RET（％）
1	女	13	VSAA	46.0	154	19.4	3.07	3.2	66	0.13	14	6
2	男	21	SAA	61.0	170	21.1	1.90	2.6	47	0.70	15	12
3	女	12	SAA	44.0	153	18.8	1.93	2.4	69	0.47	11	12
4	男	38	SAA	80.0	170	27.7	1.96	2.2	70	0.24	11	6
5	女	13	SAA	74.0	162	28.2	1.69	2.5	50	0.41	2	72
6	女	48	SAA	82.5	157	33.5	1.26	2.0	57	0.74	9	15
7	男	31	SAA	62.5	166	22.7	2.09	2.5	66	0.41	10	23
8	男	22	SAA	72.5	170	25.1	0.52	0.97	70	0.45	9	20
9	男	12	VSAA	54.0	168	19.1	2.27	2.4	60	0.13	14	4
10	男	20	SAA	53.0	158	21.2	1.60	2.9	64	0.30	8	42
11	女	15	SAA	62.0	160	24.2	2.25	2.5	52	0.25	14	4
12	女	41	SAA	77.0	163	29.0	1.04	1.5	45	0.46	9	22
13	男	8	SAA	37.5	130	22.2	2.15	2.4	70	0.25	10	16
14	女	41	SAA	67.0	162	25.5	1.19	1.9	38	0.71	4	20
15	女	25	VSAA	56.0	166	20.3	0.72	0.8	58	0.03	18	2
16	男	11	SAA	65.0	156	26.7	1.77	2.0	78	0.23	13	11

注：SAA：重型再生障碍性贫血；VSAA：极重型再生障碍性贫血；ALC：淋巴细胞绝对计数；RET：网织红细胞比值

2. 基础结构模型的建立和药代模型参数：药物浓度时间曲线见图1。p-ATG输注第1天血药浓度均快速升高，在开始输注后的16 h最大血药浓度为（22.7±20.0）mg/L；p-ATG输注期间患者血药浓度仍逐渐升高，无明显平台期，并于输注结束后第1天达峰值血药浓度；结束末次药物输注后，p-ATG血药浓度开始下降，药物清除早期较快，后期缓慢，可体内存在长达60 d。

**图1:**
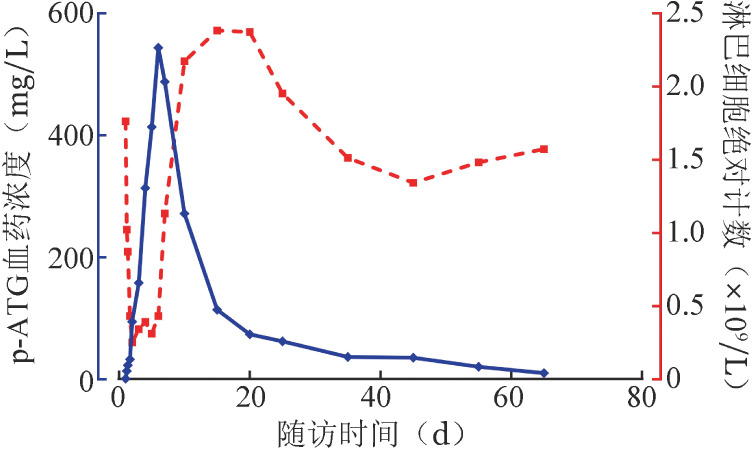
14例重型再生障碍性贫血患者p-ATG血药浓度与淋巴细胞绝对计数动态曲线 p-ATG：抗人T细胞猪免疫球蛋白

根据p-ATG在体内代谢浓度数值参数，采用DAS 3.1.4软件计算拟合，确定p-ATG药代动力学的二室模型（中央室及外周室），权重系数1/c/c。14例患者平均T_max_为（5.786±2.486）d，平均C_max_为（616±452）mg/L，平均T_1/2_为（10.479±8.242）d，平均驻留时间［MRT_（0-∞）_］为（14.43±14.02）d，分布常数（0.581±0.432）1/d，分布速率（0.352±0.282）1/d，分布相半衰期（T_1/2α_）为（4.873±3.067）d，消除常数（6.100±5.439）1/d，消除速率（0.066±0.560）1/d，平均消除相半衰期（T_1/2β_）（12.353±16.183）d，平均药物浓度时间曲线下面积［AUC_（0-t）_］为（5.807±3.236）mg/L·d。

3. ALC、有效性与药物浓度相关性：14例患者中位基线ALC为1.84（0.52～3.07）×10^9^ /L，应用p-ATG过程中，ALC迅速下降，药物浓度达峰时患者中位ALC为0.31（0.10～0.90）×10^9^/L，最低值仅为基线值的16.8%。随着ATG用药结束，药物浓度逐渐降低，ALC也逐渐复升，于用药14 d达峰值，中位水平为2.43（1.54～3.43）×10^9^/L。此后，淋巴细胞再逐渐下降至接近基线水平，第35天中位ALC为1.71（0.72～2.27）×10^9^/L（图1）。治疗后6个月，14例患者中8例有效，有效组与无效组患者AUC_（0-t）_分别为（7.50±3.26）mg/L·d、（4.50±2.18）mg/L·d，C_max_分别为（627±476）mg/L、（584±382）mg/L，有效组均高于无效组，但样本量过低，未进行假设检验。

4. p-ATG的耐受性：即刻输注不良反应主要为发热1例，皮疹1例，窦性心动过缓1例，加快糖皮质激素给药速度后患者症状缓解，未出现严重不良反应。药物治疗后7～14 d，2例患者出现血清病反应，1例为皮疹、发热，1例为水钠潴留，加大激素药物剂量均控制。

## 讨论

ATG/ALG是提取人类胸腺细胞（包括T淋巴细胞及少许B淋巴细胞）及淋巴细胞（或淋巴细胞系）免疫动物（如兔、马、猪等），产生针对人T淋巴细胞的抗体，提纯后制备的含有IgG、IgM等多克隆免疫球蛋白，是一种针对人T细胞的强免疫抑制剂。目前有关AA的发病机制国际共识认为，T淋巴细胞的功能异常是导致骨髓造血衰竭的主要机制[3]，ATG/ALG是不能选择移植治疗的SAA患者的首选治疗方案，为避免患者过度免疫抑制而发生严重感染和复发，以及ATG/ALG药物血浓度过低而疗效欠佳，因此探索SAA患者个体化的最佳ATG/ALG剂量尤为重要。

p-ATG在中国AA患者体内药代动力学特点尚未明确，而应用p-ATG治疗AA的疗效及不良反应是否与其药代动力学相关，本研究希望为临床提供数据依据。采用三抗体夹心ELISA试验法，样本处理相对简单，成本费用较低，数据可靠等优点，具有广泛开展的可能性。本研究应用p-ATG总量为100 mg/kg，分5 d应用，每日输注时间不低于12 h，C_max_为（616±452）mg/L，达到峰值为应用药物第6天，其后早期开始快速下降，第10天后开始缓慢下降，T_1/2_为10.479 d，用药后60 d仍可检测到药物残留。

研究表明p-ATG为慢代谢药物，在总剂量相同的条件下，分多日应用，避免生物制剂的单次大剂量引起的短时药物浓度过高，从而引发高热、关节痛、过敏甚至休克等药物不良反应，且分日应用可维持更好的疗效，针对本病的异常T淋巴细胞抑制更充分持久。本研究14例患者平均AUC_（0–∞）_为（5.807±3.236）mg/L·d，患者随访至治疗后6个月时判断疗效，14例患者中8例有效，有效组AUC_（0–t）_及C_max_高于无效组，但样本量过低，未进行假设检验。本研究纳入患者样本量较小，视为Ⅰ期药代动力学研究，此结果没有足够效力评价疗效影响因素，加之AA疗效影响因素众多，故无法判定药代参数与疗效相关性。未来需增加样本量进一步研究。

由于ATG的消除半衰期很长，因此临床每日应用等量ATG剂量，体内p-ATG的浓度随应用时间逐步达稳态，这是很多药物产生临床治疗作用的先决条件[5]，同时也可能是药物产生不良反应的重要原因。本研究中所有患者未出现严重不良事件，因此根据p-ATG安全耐受的结果及药代动力学特点，我们认为在SAA治疗中，按100 mg/kg分5 d静脉输注是安全的。

本研究p-ATG T_max_相较国内外报道马/猪ATG（h/p-ATG）相似[3]–[4],[6]，其T_1/2β_ 为12.353 d，短于r-ATG的21.56～29.67 d[4],[6]，与既往p-ATG在HSCT患者中的10.99 d相似[7]。用药后60 d体内检测到残存药物浓度，短于r-ATG体内残留最长90 d的清除时间[4],[6]，代谢清除相对较快。我们发现随着p-ATG体内的清除，患者淋巴细胞也很快恢复至较高水平，随着血药浓度的平稳，淋巴细胞也恢复至基线水平，这与文献报道r-ATG的IST后淋巴细胞较长时间抑制不同[8]，提示p-ATG免疫抑制强度可能略低，而与马-ATG（h-ATG）免疫抑制特征相近。

我们对SAA患者p-ATG药代特征研究表明，p-ATG是慢代谢的生物制剂，治疗过程随着药物的蓄积其浓度可快速达峰值，无明显平台期；治疗后药物清除早期较快，后期缓慢，可在体内长期残留。本研究尚不能得出药物代谢与疗效及不良反应的关系，尚需进一步研究探索。
